# Molecular Mechanisms of Bortezomib Action: Novel Evidence for the miRNA–mRNA Interaction Involvement

**DOI:** 10.3390/ijms21010350

**Published:** 2020-01-05

**Authors:** Karolina Łuczkowska, Dorota Rogińska, Zofia Ulańczyk, Edyta Paczkowska, Christian Andreas Schmidt, Bogusław Machaliński

**Affiliations:** 1 Department of General Pathology, Pomeranian Medical University, 70-111 Szczecin, Polanddoroginska@gmail.com (D.R.); edyta.paczkowska@pum.edu.pl (E.P.); 2 Department of Internal Medicine C-Haematology, and Oncology, Stem Cell Transplantation, Palliative Care, University Hospital Greifswald, Ernst-Moritz-Arndt University, 17489 Greifswald, Germany; christian.schmidt@uni-greifswald.de

**Keywords:** neuropathy, bortezomib, miRNA, microarray

## Abstract

Bortezomib is an anti-tumor agent, which inhibits 26S proteasome degrading ubiquitinated proteins. While apoptotic transcription-associated activation in response to bortezomib has been suggested, mechanisms related to its influence on post-transcriptional gene silencing mediated regulation by non-coding RNAs remain not fully elucidated. In the present study, we examined changes in global gene and miRNA expression and analyzed the identified miRNA–mRNA interactions after bortezomib exposure in human neuroblastoma cells to define pathways affected by this agent in this type of cells. Cell viability assays were performed to assess cytotoxicity of bortezomib. Global gene and miRNA expression profiles of neuroblastoma cells after 24-h incubation with bortezomib were determined using genome-wide RNA and miRNA microarray technology. Obtained results were then confirmed by qRT-PCR and Western blot. Further bioinformatical analysis was performed to identify affected biological processes and pathways. In total, 719 genes and 28 miRNAs were downregulated, and 319 genes and 61 miRNAs were upregulated in neuroblastoma cells treated with bortezomib. Possible interactions between dysregulated miRNA/mRNA, which could be linked to bortezomib-induced neurotoxicity, affect neurogenesis, cellular calcium transport, and neuron death. Bortezomib might exert toxic effects on neuroblastoma cells and regulate miRNA–mRNA interactions influencing vital cellular functions. Further studies on the role of specific miRNA–mRNA interactions are needed to elucidate mechanisms of bortezomib action.

## 1. Introduction

The ubiquitin/proteasome system (UPS) is responsible for the majority of intracellular eukaryotic protein turnover, and its coordinated function is essential for many cellular processes, including cell growth, differentiation, activation, and signalling [1]. Because the UPS is a crucial regulator of cell proliferation and apoptosis, therapeutic strategies to regulate its role could potentially restore stable cellular homeostasis in some cancer patients [2]. The first proteasome inhibitor approved by the US FDA for the treatment of multiple myeloma (MM) patients was bortezomib, a low-molecular-weight, water-soluble dipeptide boronic acid derivative that binds with high affinity to the proteasome and show slow dissociation [3]. Bortezomib has also shown promise as possible antitumor agent in preclinical studies of tumours such as ovarian cancer, lung cancer, pancreatic cancer, prostate cancer, melanoma, glioblastoma, and most recently in MYC-driven neuroblastoma [4,5,6]. Bortezomib is a reversible inhibitor of the chymotrypsin-like activity of the 26S proteasome degrading ubiquitinated proteins [7]. This leads to the accumulation of misfolded and functional proteins in the endoplasmatic reticulum (ER) and cytosol of cells followed by ER overload, generation of excess oxygen species as well as a functional disorder of intracellular proteins, and eventually cell death [8].

Even though inhibitory action of bortezomib is rather clearly established, the downstream mechanisms of its activity remain not fully understood [9]; it is therefore of great interest to understand the mechanism of action of bortezomib. One of the main side effects of bortezomib treatment, occurring in up to 40% MM patients, remains peripheral neuropathy, which has been linked with neurotoxicity [10,11]. It has been hypothesized that specific neurotoxic action of bortezomib is through transient release of intracellular calcium stores, leading to mitochondrial calcium influx and caspase-induced apoptosis [7]. While apoptotic transcription-associated activation in response to bortezomib has been suggested, mechanisms and new insights, particularly in the post-transcriptional gene-silencing-mediated regulation, remain largely unexplained [12]. MicroRNAs (miRNAs, miRs), 20–22 nucleotide long non-coding RNA molecules, have recently emerged as master regulators of various genes and their role in bortezomib action is also suggested [9,12,13,14]. MiRNAs regulate gene expression at the post-transcriptional level and are involved in almost every biological process, including cell proliferation, apoptosis, as well as disease-associated processes [15]. Differentially expressed miRNAs have been reported in a number of diseases [16] and as a response to drug use [17], however to date, the wide panel of mRNAs/miRNAs has not been tested in bortezomib-treated cells.

In order to better understand the underlying molecular mechanisms of bortezomib action in a neural environment, in the present study, we investigated global mRNA and miRNA expression in bortezomib-treated neuroblastoma cells and analyzed the identified miRNA–mRNA target interaction.

## 2. Results

### 2.1. SH-SY5Y Cells Viability

**Figure 1:**
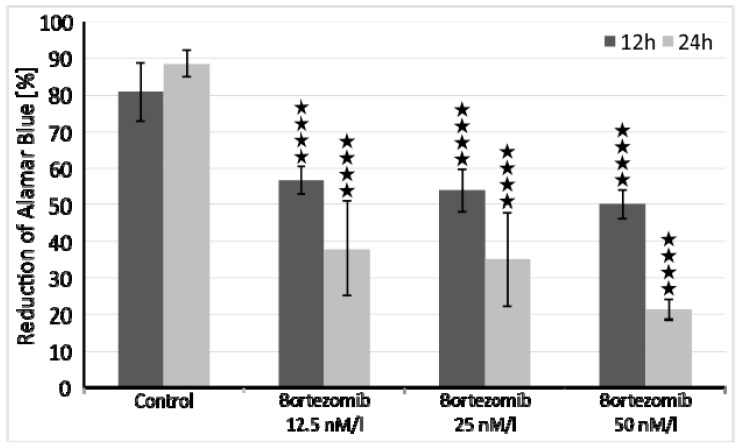
Cell viability assays with Alamar Blue. Data are presented as a percentage of Alamar Blue reduction, observed in 12 or 24-h period. **** *p* < 0.0001 compared with the control cells.

Bortezomib has proven to be adequate in 24-h incubation period in concentration of 50 nM/L (Figure 1), where it showed higher percentage of reduced Alamar blue (lower % of viable cells) both versus control and preceding dose (25 nM/L).

### 2.2. Gene Expression Profile in Neural Cells

**Figure 2:**
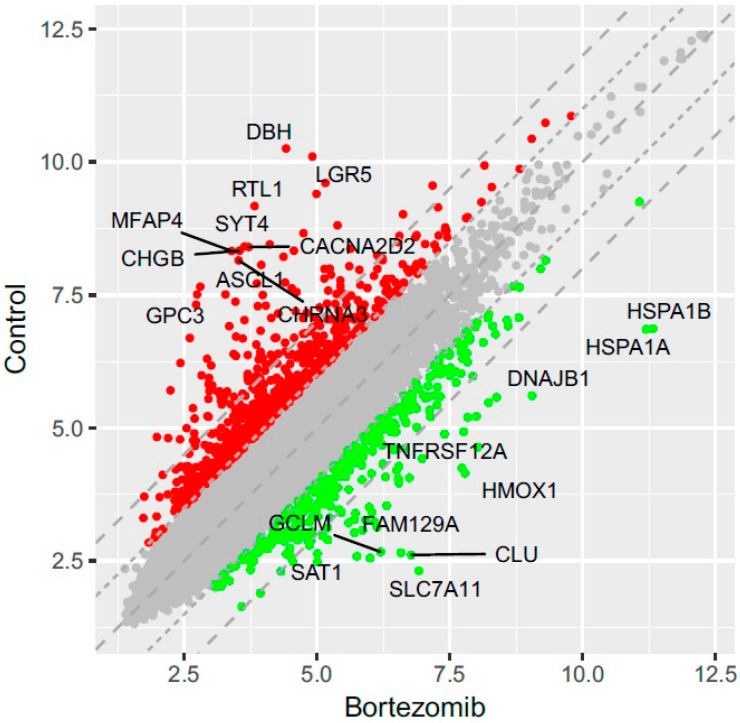
The scatterplot of global gene expression in bortezomib-treated SH-SY5Y cells compared to the control cells. Red points show downregulated genes (at least 2-fold change, *p* < 0.05), green points show upregulated genes (at least 2-fold change, *p* < 0.05). The graph also comprises the names of genes with the largest change in expression.

**Figure 3:**
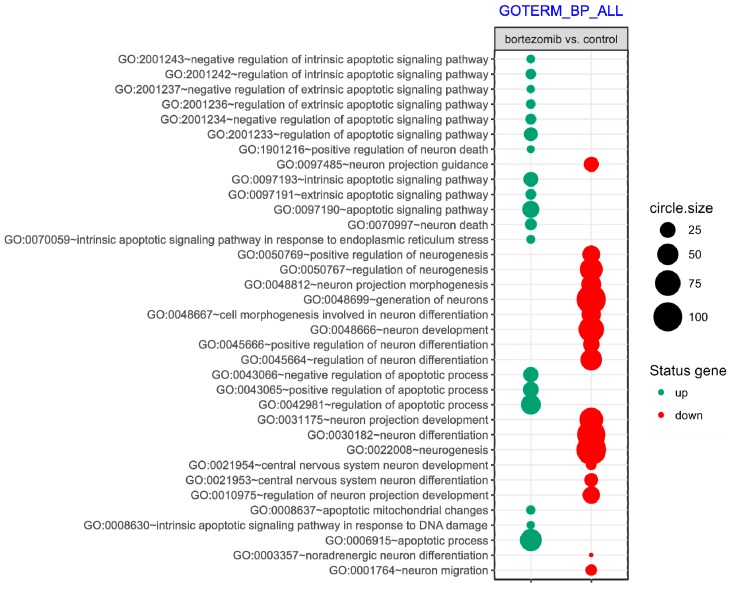
The bubble plot of overrepresented biological processes involved in apoptosis and neurogenesis assigned according to gene ontology (GO) classification in bortezomib-treated SH-SY5Y cells compared to the control cells The groups of genes fulfilling criteria: Adjusted *p* < 0.05, method = Benjamini, and minimum number of genes per group = 5, are presented. Bubble size indicates the number of genes represented in corresponding annotation. The up- and downregulation is indicated by colors, with green corresponding to upregulation and red corresponding to downregulation.

Microarray analysis revealed that 719 genes were at least 2-fold downregulated and 319 genes were at least 2-fold upregulated in neuroblastoma cells treated with bortezomib when compared to control cells (Figure 2). The differentially expressed genes were classified according to the gene ontology (GO) classification of biological processes. Analysis of functional annotations identified 16 apoptotic processes, that were significantly upregulated (e.g., regulation of apoptotic signaling pathway; intrinsic apoptotic signaling pathway; apoptotic signaling pathway; or positive regulation of apoptotic process) and 19 neurogenesis processes that were significantly downregulated (e.g., regulation of neurogenesis; generation of neurons; neuron development; regulation of neuron differentiation; regulation of neuron projection development; central nervous system neuron differentiation). The bubble diagram illustrating the overrepresented terms is shown in Figure 3.

Given the ability of bortezomib to exert neurotoxicity in patients, we were interested in biological processes concerning nervous system. Comparing the complex gene dataset in bortezomib-treated cells to that of control cells, we identified several genes involved in neurogenesis (fold change between −28.9 and −2). Detailed values of gene expression differences for selected genes are provided in Table 1. Listed genes are responsible for processes essential for neural tissue function such as neuronal cells differentiation, migration, regeneration, protection, axon guidance, synapse formation, and proliferation.

Dysregulation of Ca^2+^ homeostasis can also contribute to disorders of the nervous system and lead to neuropathy symptoms. We observed five downregulated genes (fold change between −25.7 and −2) involved in the calcium transport critical to neuronal conduction (Table 2). Moreover, 14 upregulated genes in bortezomib-treated cells are associated with neuron death (fold change between 21.9 and 2) (Table 3). Further analysis of differentially expressed genes in neural cells after bortezomib exposure revealed genes involved in the regulation of apoptosis (Table 4 and Table 5).

#### Validation of Dysregulated mRNAs

**Figure 4:**
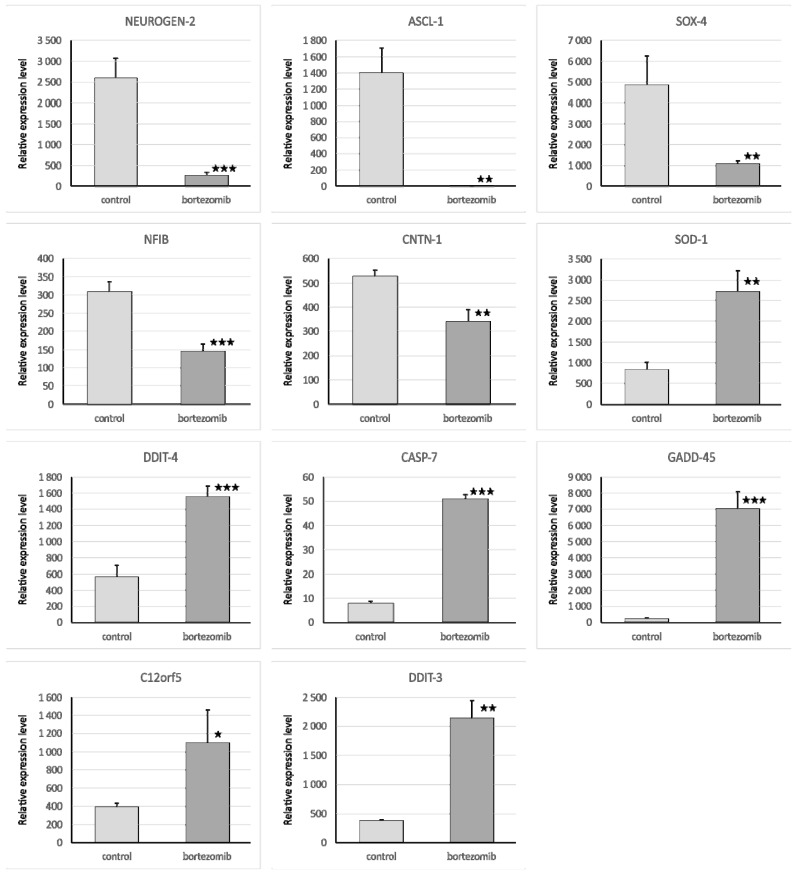
Real-time quantitation of selected genes *Neurogen2, NFIB, CNTN1, SOD1, DDIT4, CASP7, SOX4, GADD45A, C12orf5, ASCL1,* and *DDTI3* in SH-SY5Y cells treated with bortezomib and in the control cells. Data are presented as the mean ± SD (*n* = 3). * *p* < 0.05; ** *p* < 0.01; *** *p* < 0.001 compared with the control cells.

**Figure 5:**
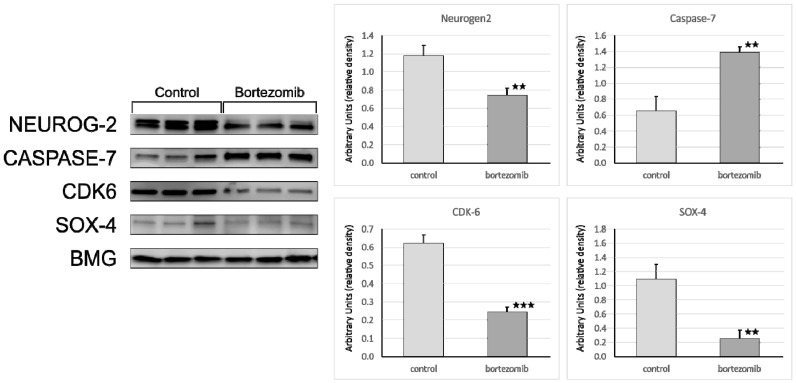
Western blot quantitation of selected proteins: Neurogenin2, Caspase7, CDK6, and SOX4 in SH-SY5Y cells treated with bortezomib and in the control cells. Data are presented as the mean ± SD (*n* = 3). * *p* < 0.05; ** *p* < 0,01; *** *p* < 0.001 compared with the control cells.

We selected significantly dysregulated genes (*Neurogen2, NFIB, CNTN1, SOD1, DDIT4, CASP7, SOX4, GADD45A, C12orf5*, *ASCL1, DDTI3*) to perform qRT-PCR experiments. In all cases, the results of qRT-PCR confirmed the differences in expression of selected mRNAs revealed by the global gene expression analysis (Figure 4). Furthermore, we also demonstrated the level of selected proteins (NEUROGEN2, SOX4, CDK6, Casp7) using Western blot analysis. Similarly, the results of selected genes expression on protein level obtained by Western blot confirmed the significant differences revealed by microarray assay (Figure 5).

### 2.3. MiRNAs Expression Profile in Neural Cells

**Figure 6:**
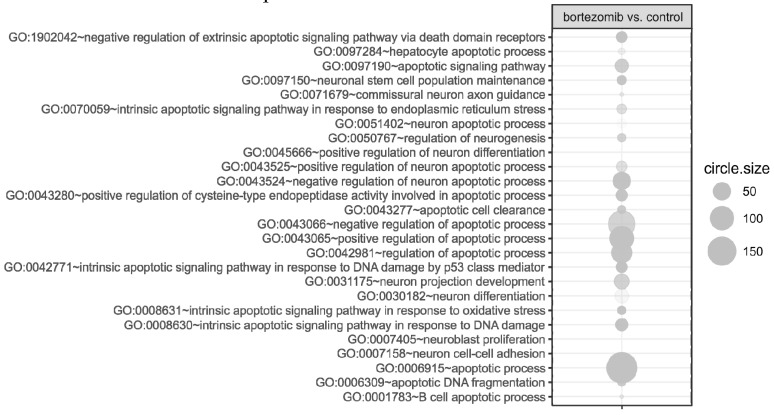
The bubble plot of overrepresented biological process involved in apoptosis and neurogenesis assigned according to gene ontology (GO) classification in bortezomib-treated SH-SY5Y cells compared to the control cells Groups of genes fulfilling criteria: Adjusted *p* < 0.05, method  =  Benjamini, and minimum number of miRNAs per group  =  5, are presented. Bubble size indicates the number of miRNAs represented in corresponding annotation.

Since miRNA regulates gene expression, we performed combined analysis comparing the results of miRNA expression microarray with the data obtained from the genomic microarray’s analysis. Table 6, Table 7, Table 8 and Table 9 present fold change for individual miRNAs and their target genes including directions of gene expression changes (up or down). Aberrantly expressed miRNAs were assigned to specific biological processes according to the gene ontology (GO) classification. Figure 6 presents a diagram illustrating the overrepresented terms. Analysis of functional annotations identified 16 apoptotic processes (e.g., apoptotic signaling pathway, neuron apoptotic process, regulation of apoptotic process, apoptotic DNA fragmentation) and 10 neurogenesis associated processes (e.g., regulation of neurogenesis, neuron projection development, neuron differentiation) that were altered in bortezomib-treated neural cells compared to control cells.

**Figure 7:**
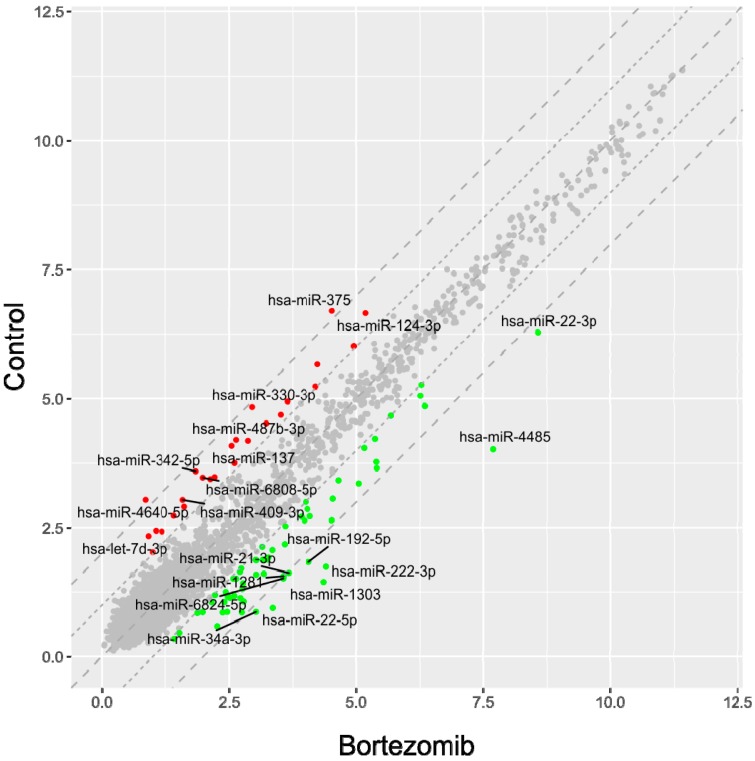
The scatterplot graph of global miRNAs expression in bortezomib-treated SH-SY5Y cells compared to the control cells. Red points show downregulated miRNAs (at least 2-fold change, *p* < 0.05), green points show upregulated miRNAs (at least 2-fold change, *p* < 0.05). The graph also comprises the names of miRNAs with the largest change in expression.

The result of miRNA expression analysis showed 61 significantly upregulated miRNAs (at least 2-fold) and 28 downregulated (at least 2-fold) in bortezomib-treated neural cells as compared to control cells. Figure 7 presents scatter plot graph of global miRNAs expression in neuroblastoma cells after treatment with bortezomib.

Next, we analyzed miRNAs that regulate expression of genes involved in neurogenesis and apoptosis in neural cells after bortezomib, we found 22 upregulated miRNAs, which reduce expression of 25 genes involved in neurogenesis (Table 6).

Further analysis indicated that 23 significantly upregulated miRNAs regulate expression of 30 genes involved in anti-apoptotic processes and lead to decreased expression of these genes in bortezomib-treated neural cells. Two from all of the downregulated miRNAs (fold change ≥2) regulate the expression of genes involved in neuron death, while nine upregulated miRNAs (fold change ≥2) alter the expression of 17 genes involved in proapoptotic processes (Table 7, Table 8 and Table 9).

To summarize, bortezomib induced aberrant miRNAs expression and altered miRNA–mRNA interactions in neural cells enhancing apoptosis and neuron death as well as suppressing neurogenesis.

#### Validation of Dysregulated miRNAs

**Figure 8:**
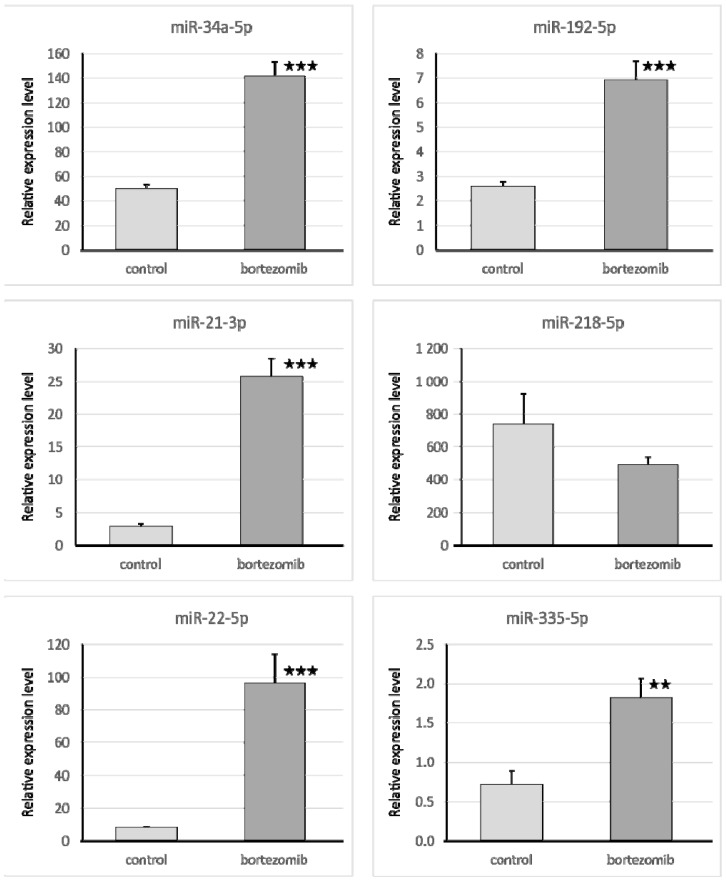
Real-time quantitation of selected miRNAs (34a-5p; 192-5p; 21-3p; 218-5p) in SH-SY5Y cells treated with bortezomib and in control cells. Data are presented as the mean ± SD (*n* = 3). * *p* < 0.05; ** *p* < 0.01; *** *p* < 0.001 compared with the control cells.

We selected six significantly dysregulated miRNAs to perform qRT-PCR experiments (miR34a-5p; miR192-5p; miR21-3p; miR218-5p; miR22-5p; miR335-5p). In all cases, the results of qRT-PCR confirmed the differences in expression of selected miRNAs revealed by the global gene expression analysis (Figure 8).

## 3. Discussion

Bortezomib action, owing to its powerful inhibiting proteasome activity, has been correlated with inhibition of anti-apoptotic proteins, p53 stabilization, cell cycle progression disruption, and other critical cellular processes [18,19]. Using global mRNA and miRNA expression analysis, we demonstrate for the first time that bortezomib exposure leads to dysregulation of a number of genes and miRNAs associated with neurogenesis and apoptosis in neural cells.

First, we looked into neurogenesis as a process that may be affected by bortezomib use. Several important genes were downregulated, including *NEUROG2, ASCL1, SYT4,* however no specific correlations with miRNAs were found. The upregulation of miR-1303 could be attributed to the nature of neuroblastoma cells, as miR-1303 has been shown to promote the proliferation of SH-SY5Y cells; several studies showed that miR-1303 is upregulated in neuroblastoma cells and tissues [20]. miR-1303 decreases doublecortin (*DCX*), which plays an important role in neurogenesis and its deletion causes severe morphologic defects and delayed migration along the rostral migratory stream [21]. Another significantly upregulated miRNA was miR-21-3p, which decreases *NCAM2, ELMO1,* and *GPAM* expression. Contrary to our findings, Wang et al. have reported that bortezomib decreases level of miRNA-21, which is considered as “oncomir”, since it suppresses the expression of several apoptotic and tumor suppressor genes leading to cell proliferation, migration, invasion, angiogenesis, and metastasis as well as inhibition of apoptosis in human myeloma cell lines [14,22]. Our results might suggest that miR-21 dysregulation contributes to neuron death, because of important roles of miR-21 targets, which it downregulates. *NCAM2* is primarily expressed in the brain, where it stimulates neurite outgrowth and facilitates dendritic and axonal compartmentalization [23]. *ELMO1* protects endothelial cells from apoptosis [24] and it is also an important factor for glomerular protection and renal cell survival via decreasing apoptosis, especially under diabetic conditions [25].

We also found correlations between overexpression of miRNAs that target genes essential in neuronal development and remodelling: miR-6824, -335, and -1468 all target *SOX4*, which participates in Schwann cell myelination [26], whereas miR-6836 targets *TFAP2B*, which acts in the specification of GABAergic interneurons in developing cerebellum [27]. Interestingly, *MYC*-miR34a axis, in which miR-34a apoptotic actions are tightly regulated by *MYC* and this can lead to drug resistance [28], has somehow been confirmed by our study, however the role of miR-4668 (which was also upregulated and targets *MYC*) in these interactions remains unclear. The overexpression of miR-3192 and consequent downregulation of *CHRNB2*, which ensures communication between neurons, could be another possible neurotoxicity mechanism [7], due to disruption of axonal transport processes. Additionally, *NCAM* (fold change -5.59), which is a target for miR-21-3p, activates several signal transduction pathways or genes such as glial cell line-derived neurotrophic factor (*GDNF*), fibroblast growth factor receptor (*FGFR*) [29]. This finding may support hypothesis that reduction of neurotrophins production is also involved in bortezomib action. Several of miRNAs, which showed altered expression in this study, have been described in MM patients before, including miR-21 and miR-222 [30,31], but a correlation between their dysregulation and bortezomib neurotoxicity is yet to be found.

Bortezomib cytotoxicity is also attributed to mitochondrial-mediated dysregulation of calcium homeostasis [7]. The mitochondrial Ca^2+^ overload is one of the pro-apoptotic ways to induce the swelling of mitochondria, with perturbation or rupture of the outer membrane followed by a release of mitochondrial apoptotic factors into the cytosol [32]. Disruption of intracellular calcium homeostasis may be an emerging mechanism involved in drug-induced neuroapoptosis [33,34]. Landowski et al. demonstrated that the cytotoxic action of bortezomib is mediated through a transient release of intracellular calcium store leading to mitochondrial calcium inflow and apoptosis induced by caspase-12 activation [35,36]. In the present study, we observed very low levels of important genes encoding calcium channels (*CACNA2D2, CACNG4*) and regulating the calcium level (*ITPR1*) (Table 2), although no specific miRNA–mRNA interactions were found. The downregulation of these genes may have a significant impact on neural function as they regulate intracellular calcium release, which is fundamental to neural cells’ functions including neuronal excitability, neurite outgrowth, neurotransmitter release, mitochondrial energy production, and cell fate [37]. These data support the hypothesis that bortezomib can induce neurotoxicity by disrupting intracellular calcium homeostasis in neural cells.

Another possibility is that bortezomib triggers oxidative stress, which contributes to proapoptotic effects and gene expression patterns reprogramming in nerves. In the present study, we observed a significant upregulation of genes engaged in apoptosis, especially related to neuron death. Among the most strongly upregulated genes were clusterin (*CLU*), heme oxygenase (decycling) 1 (*HMOX1*), caspase 7 (apoptosis-related cysteine peptidase, *CASP7*), and DNA-damage-inducible transcript 3 (*DDIT3*). Products of these genes are synthesized in response to cellular stress and appear in cells undergoing apoptosis [38,39,40,41]. Larner et al. have demonstrated that *CASP7* is significantly upregulated and activated in both neurons and astrocytes after traumatic brain injury (TBI) in rats and may play a role in the cell death in TBI in patients [42]. An interaction between miR-124 and interleukin 6 receptor (*IL6R*), which was observed in our study, could also contribute to bortezomib action, since *IL6R* is a linking factor between several important pathways including NF-κB, MAPK, and JAK/STAT, which take part in a variety of cellular responses, including cell survival, inflammation, and differentiation [43]. *SQSTM1,* which in our study was significantly upregulated by miR-218, is a useful indicator of autophagy—it accumulates in autophagic-defective tumor cells and decreases when autophagy is completed. *SQSTM1* upregulation observed in this study and thus enhanced autophagy might also contribute to bortezomib neurotoxicity, also by acting on ERK phosphorylation [44]. The role of *IGF1R* in bortezomib response is also suggested [45]; in our study, miR-4423 and miR-551 significantly affected expression of this gene. One of the most prominently downregulated miRNAs in this study was miR6880-5p, responsible for downregulation of proapoptotic gene *C12orf5,* which promotes neuron death (Table 8). In case of decreased miR6880-5p, *C12orf5* expression is no longer downregulated, which was also seen in our study, as we observed upregulation of *C12orf5.* In the same manner, downregulation of miR-26b-5p causes increased expression of *DDIT4, HSPD1, ADAM17, DDIT4, HSPD1, EPHA2,* and *RNF216*. These genes promote apoptosis, DNA damage, and neuron death. Therefore, the induction of neuronal cell apoptosis may represent a relevant step in bortezomib action.

In this study, we focused on miRNA/mRNA interactions, however there are several reports describing dysregulation of genes/miRNAs without exact interactions between them, but which are also relevant to our results. Liu et. al. have demonstrated that treatment with bortezomib significantly increased *ATF3*, *c-JUN,* and *CCL2* expression in DRG in rat model [46]. Blockage of c-Jun/ATF3-CCL2 signaling pathway by siRNA has been shown to attenuate the mechanical allodynia induced by bortezomib. These findings suggested that the upregulation of *CCL2* via enhanced interaction between *c-Jun* and *ATF3* contributes to bortezomib-induced mechanical allodynia [46]. Interestingly, it has been demonstrated that the treatment with paclitaxel significantly increases the *ATF3* expression in rat DRG neurons, suggesting that *ATF3* might be involved in the chemotherapy-induced neurotoxicity [47]. In our study, we observed increased expression of *ATF3* (fold change = 6.37) and *JUN* (fold change = 2.74) in neural cells treated with bortezomib compared with control cells. However, the expression of *CCL2* was unchanged after bortezomib exposure. It may result from relatively short incubation time in setting of our study or different model of experiment.

### Potential Study Limitations

Overall, our study gave some interesting results, but did not lack some drawbacks. The first limitation is only one time point and dose at which array experiment was conducted. Additionally, we did not perform functional validation assays to further test the effect of mRNA-miRNA interactions on neurogenesis, neuron death, and cellular calcium transport. We believe that different drug doses, time points, and cellular models could be of great importance to further elucidate the underlying mechanism of bortezomib action. Due to the basic and preliminary nature of this study, we should be cautious about ascribing cause and effect of bortezomib exposure on mRNA-miRNA interactions and neurotoxicity.

## 4. Materials and Methods

### 4.1. Cell Culture and Incubation

SH-SY5Y neuroblastoma cells (human, ECACC; Sigma Aldrich, St. Louis, MO, USA) were used in this study. Selected cell line is frequently used as a reliable model for studying neurotoxicity of drugs [48,49]. SH-SY5Y cells were incubated at a density of 4 × 10^6^ cells/well in 6-well culture plates in Ham’s F-12 Nutrient Mixture (Thermo Fisher, Waltham, MA, USA) and minimum essential medium (MEM) (Sigma Aldrich, St. Louis, MO, USA) (mixed in ratio 1:1) medium supplemented with penicillin (100 U/mL), streptomycin (100 µg/mL), and L-glutamine (2 mM) without fetal bovine serum at 37 °C in saturated humidity atmosphere containing 5% CO_2_. Cells were not treated (control) or treated with bortezomib 50 nM/l (Cell Signalling Technology, Danvers, MA, USA) and collected after 24 h of incubation [50,51]. Cells were then subjected to mRNA, miRNA, and protein isolation.

### 4.2. Cells Viability

The optimal dose of bortezomib for this experiment was based on the already published data [50,51]. However, we performed additional cell viability assays to assess cytotoxicity of tested drug. SH-SY5Y cells were incubated at a density of 1x10^4^ cells/well in two 96-well culture plates (medium as in 2.1), first plate for 12 and second plate for 24 hours at 37 °C in saturated humidity atmosphere containing 5% CO_2_. Each 8-well row was treated with increasing concentrations of bortezomib (12.5 nM/l, 25 nM/l, 50 nM/l) and cell viability was measured with the Alamar Blue mitochondrial dye conversion assay (Thermo Fisher, Waltham, MA, USA) [50].

### 4.3. RNA and Protein Extraction

RNA and protein were isolated from three separate cell incubations for each group. Total RNA was isolated from SH-SY5Y cells (1 × 10^6^) using the mirVana™ miRNA Isolation Kit (Thermo Fisher, Waltham, MA, USA) following the manufacturer’s instructions. The kit allows the isolation of RNA and miRNA simultaneously. Concentration and quality of the obtained RNA were assessed by Epoch spectrophotometer (Biotek, Winooski, VT, USA). SH-SY5Y cells (3 × 10^6^) exposed to the dose of bortezomib and control cells were lysed for 10 min on ice in M-Per lysing buffer (Pierce, Rockford, IL, USA) containing protease and phosphatase inhibitors (Sigma Aldrich, St. Louis, MO, USA) (10 µg/ml leupeptin, 10 µg/mL aprotinin, 1 µg/mL pepstatin A, 1 mM sodium fluoride, and 2 mM Na_3_VO_4_). The measurement of total protein was performed using the Bradford method.

### 4.4. Affymetrix GeneChip Microarray and Data Analysis

Total RNA isolated from three separate cell incubations were pooled to generate one sample for subsequent experimental procedures. A sense-strand cDNA generated from the total RNA using an Ambion WT Expression Kit (Thermo Fisher Scientific, Waltham, MA, USA) was fragmented and labeled using the GeneChip WT Terminal Labeling Kit (Affymetrix, Santa Clara, CA, USA) and next hybridized onto an Affymetrix Human Gene 2.1 ST Array Strip. The hybridization and subsequent fluidics and scanning steps were carried out with an Affymetrix GeneAtlas System, with designated software (Affymetrix, Santa Clara, CA, USA). All analyses were performed using BioConductor software based on the statistical R programming language. The robust multiarray average (RMA) normalization algorithm implemented in the “Affy” library was used for normalization, background correction, and calculation of the expression levels of all of the examined genes [52].

### 4.5. Affymetrix GeneChip miRNA Microarray and Data Analysis

Total RNA-enrichment in miRNAs isolated from three separate cell incubations were pooled to generate one sample for subsequent experimental procedures. The procedure was started with a poly (A) tailing reaction followed by ligation of the biotinylated signal molecule to the target RNA. The next step was sample hybridization onto an Affymetrix miRNA 4.1 Array Strip (Affymetrix, Santa Clara, CA, USA). The last step was streptavidin-PE addition and array scanning with Affymetrix GeneAtlas system (Affymetrix, Santa Clara, CA, USA). Analysis of microarray data was performed using BioConductor, as described earlier in 2.3. [52]. Normalized data were combined with “pd.mirna.4.1” description file, containing, among others, names, types, and sequences of miRNAs. Differential expression was determined by applying the linear models for microarray data implemented in the “limma” library [53]. Normalized miRNA expression datasets were visualized on scatterplots with relation to determined cut-off criteria (fold change > abs.2).

The list of experimentally validated miRNA target genes was downloaded from miRTarBase—a database of mRNA–target interactions. Only targets for differentially expressed miRNA were subtracted from the whole human miRNA-target dataset. Target gene list from each of comparisons was subjected to functional annotation and clusterization using DAVID (Database for Annotation, Visualization, and Integrated Discovery) [54]. Target symbols of differentially expressed miRNA were uploaded to DAVID by the “RDAVIDWebService” BioConductor library [55], where targets were assigned to relevant gene ontology (GO) terms. GO ontological groups participating in the apoptosis and neurogenesis processes were visualized using bubble plot.

miRNA–target genes interaction network was performed using “networkD3” library. Taking into consideration that miRNA mostly act as repressors of target genes transcription, only target genes that negatively correlated with miRNA were visualized (if the expression of miRNA was increased, the expression of its targets should be decreased and vice versa). With such an assumption, an interaction network was performed for all of differentially expressed miRNA and target genes, as well as for miRNA and targets participating in apoptosis and neurogenesis.

### 4.6. DAVID

DAVID Bioinformatics Resources (Database for Annotation, Visualization, and Integrated Discovery) at http://david.abcc.ncifcrf.gov is functional annotation and enrichment analysis. This database is an integrated tool biological knowledgebase and tools to systematically extract substantial biological terms associated with a given gene list. Functional annotation charts generated by DAVID with overrepresented gene annotations are shown as bubble plots from the BACA BioConductor package (https://cran.r-project.org/web/packages/BACA/BACA.pdf). Bubble plots were generated with the following criteria: *p* value < 0.5, adjusted method = Benjamini, and minimal number of genes per group = 5. Groups of genes fulfilling the mentioned criteria are presented in a graph in which the bubble size indicates the number of genes represented in the corresponding annotation and the condition of these genes in terms of their down- and upregulation [54,55].

### 4.7. qRT-PCR for Validation of Expression of Selected mRNA/miRNA

The qRT-PCR reaction was used to evaluate the expression of genes and miRNA. We selected significantly dysregulated (fold change > 2.0) mRNA/miRNA for validation of array experiments. Reaction was performed on a Bio-Rad CFX96 Real-Time PCR Detection System (Bio-Rad Inc., Hercules, CA, USA). All primers were purchased from Laboratory of DNA Sequencing and Oligonucleotide Synthesis, Institute of Biochemistry and Biophysics, Polish Academy of Sciences, Warsaw, Poland. The qRT-PCR program consisted of 10-min initial denaturation followed by a 3-step amplification program: Denaturation at 95 °C for 15 s, annealing at 56 °C for 5 s, and extension at 72 °C for 10 s. Specificity of reaction products was checked by determination of melting points (0.1 °C/s transition rate). The relative gene expression was quantified using the comparative Ct method (2ΔCt, where ΔCt = [Ct of target genes] − [Ct of endogenous control gene]).

#### 4.7.1. Validation of Gene Expression

Isolated mRNA (0.1 µg) was reverse transcribed using the First Strand cDNA Synthesis Kit (Thermo Fisher Scientific, Waltham, MA, USA). The reaction mixture for qRT-PCR consisted of 7.5 μL of SYBR Green PCR Master Mix; 1.5 μL cDNA template; 4.2 μL Nuclease-Free Water; and 1.8 μL specific primers (0.9 μL forward primer and 0.9 μL reverse primer). The primers were designed by BLAST, and *BMG* was used as an endogenous control gene.

#### 4.7.2. Validation of Selected miRNA Expression

Isolated miRNA (0.1 µg) was reverse transcribed using qScript microRNA cDNA Synthesis Kit (Quanta Biosciences, Beverly, MA, USA), which contains all necessary components for qRT-PCR. The reaction mixture for qRT-PCR contained: 5 μL PerfeCTa SYBR Green SuperMix; 0.2 μL microRNA specific primer; 0.2 μL PerfeCTa Universal PCR Primer, 1 μL microRNA cDNA; 4.6 μL Nuclease-Free Water. The primers were designed by miRPrimer, and miR93 was used as an endogenous control gene.

### 4.8. Western Blot

The protein samples were applied (20 µg/well) and separated on a 4%–20% sodium dodecyl sulfate polyacrylamide gel electrophoresis (SDS-PAGE, miniPROTEAN II electrophoresis system, Bio-Rad) and next transferred to a 0.2 µm polyvinylidene fluoride (PVDF) membrane (Bio-Rad Inc., Hercules, CA, USA). After 2 h blocking in 3% BSA at RT conditions, the membrane was probed with a specific primary antibody as follows: Anti-Neurogenin2 (at 1:1000 dilution) (Abcam, Cambridge, UK; cat. number ab109172); anti-Cdk6 (at 1:750 dilution) (Abcam, Cambridge, UK; cat. number ab124821); anti-SOX4 (at 1:100 dilution) (Abcam, Cambridge, UK; cat. number ab80261); anti-Caspase-7 (at 1:750 dilution) (Abcam, Cambridge, UK; cat. number ab69540) and incubated overnight at 4 °C. To visualize the bands secondary antibody (specific to the primary antibody used in the previous step) conjugated with horseradish peroxidase (HRP) were used. Chemiluminescence detection was performed using the ECL Advance Detection Kit (Amersham Life Sciences, Buckinghamshire, UK), and the bands were visualized with a UVP camera (Gel DOC-It Imaging system, Bio-Rad). The ImageJ software ver. 1.8.0 (NIH, WI, USA) was used to determine the background-subtracted density of the bands. The relative protein expression levels of Neurog2, SOX4, Caspase7, and CDK6 were quantified in comparison to those of BMG.

### 4.9. Statistical Methods

The arithmetical means and standard deviations were calculated using MS Excel 2016. Comparisons of parameters between two groups were made using unpaired Student’s t-test and *p*-value < 0.05 was considered statistically significant. ANOVA with post-hoc Tukey’s test was used to calculate statistical significance in cell viability tests, *p*-value < 0.05 was considered significant.

## 5. Conclusions

Our current study provided a basic evidence that bortezomib might exert toxic effects on neural cells, induce aberrant miRNAs expression, and regulate miRNA–mRNA interactions affecting neurogenesis, cellular calcium transport, and apoptosis. We have shown that bortezomib exhibits a significant inhibitory effect on cell proliferation of human neuroblastoma cells in vitro and could affect other vital cellular processes. These mechanisms could underlie the advantages of using bortezomib for neuroblastoma treatment. Our promising results provide a rationale to make further investigations on effectiveness, mechanisms of action, and use of bortezomib in the treatment of neuroblastoma. Since miRNAs–mRNAs interactions are remarkably complex, we probably just touched the tip of an iceberg in this study and there is still a lot to learn. Further studies on the role of specific miRNAs–mRNAs interactions are needed to elucidate bortezomib action in neural environment and its neurotoxicity.

## Figures and Tables

**Table 1:** The list of 15 most downregulated genes involved in neurogenesis in bortezomib-treated SH-SY5Y cells compared to controls. Gene function according to NCBI Gene database.

Gene Symbol	Gene Name	Gene Function	Fold Change
*ASCL1*	achaete-scute family bHLH transcription factor 1	transcription factor, plays a role in the neuronal commitment and differentiation and in the generation of olfactory and autonomic neurons	−28.93
*SYT4*	synaptotagmin IV	multi-functional regulator of Ca2+-dependent exocytosis	−27.37
*CHRNA3*	cholinergic receptor, nicotinic, alpha 3 (neuronal)	ligand-gated ion channel that likely plays a role in neurotransmission	−24.75
*NEUROG2*	neurogenin 2	plays a role in the differentiation and survival of midbrain dopaminergic neurons	−16.98
*SERPINF1*	serpin peptidase inhibitor, clade F	neurotrophic factor involved in neuronal differentiation	−15.03
*RET*	ret proto-oncogene	transmembrane receptor and member of the tyrosine protein kinase family, binds GDNF	−14.43
*CNTN1*	contactin 1	formation of axon connections in the developing nervous system	−13.86
*NNAT*	neuronatin	forming and maintaining the structure of the nervous system	−11.48
*ISL1*	ISL LIM homeobox 1	required for motor neuron generation	−10.74
*SLIT3*	slit homolog 3 (Drosophila)	interacts with roundabout homolog receptors to effect cell migration	−10.10
*BAI3*	brain-specific angiogenesis inhibitor 3	brain-specific angiogenesis inhibitor	−8.98
*EFNB2*	ephrin-B2	mediates developmental events, especially in the nervous system and in erythropoiesis	−8.96
*PHOX2B*	paired-like homeobox 2b	transcription factor involved in the development of several major noradrenergic neuron populations and the determination of neurotransmitter phenotype	−7.38
*CDK6*	cyclin-dependent kinase 6	important for cell cycle G1 phase progression and G1/S transition	−6.75
*DPYSL3*	dihydropyrimidinase-like 3	regulates cell adhesion and migration	−6.68

**Table 2:** The list of most downregulated genes involved in Ca^2+^ transport in bortezomib-treated SH-SY5Y cells compared to controls. Gene function according to NCBI Gene database.

Gene Symbol	Gene Name	Gene Function	Fold Change
*CACNA2D2*	calcium channel, voltage-dependent, alpha 2/delta subunit 2	modulates calcium currents and channel activation/inactivation kinetics	−25.76
*CACNG4*	calcium channel, voltage-dependent, gamma subunit 4	regulates both trafficking and channel gating of the AMPA receptors	−21.86
*CACNA2D1*	calcium channel, voltage-dependent, alpha 2/delta subunit 1	cleaved into multiple chains that comprise the alpha-2 and delta subunits of the voltage-dependent calcium channel complex	−3.89
*ORAI3*	ORAI calcium release-activated calcium modulator 3	forms an ER Ca(2+) leak channel that is limiting the overloading with Ca(2+) of the ER store	−2.23
*ITPR1*	inositol 1,4,5-trisphosphate receptor, type 1	mediates calcium release from the endoplasmic reticulum	−2.91

**Table 3:** The list of 10 most upregulated genes involved in neuron death process in bortezomib-treated SH-SY5Y cells compared to controls. Gene function according to NCBI Gene database.

Gene Symbol	Gene Name	Gene Function	Fold Change
*CLU*	clusterin	involved in several basic biological events such as cell death, tumor progression, and neurodegenerative disorders	17.86
*HMOX1*	heme oxygenase (decycling) 1	cleaves heme to form biliverdin	12.45
*GCLM*	glutamate-cysteine ligase, modifier subunit	first rate limiting enzyme of glutathione synthesis	11.52
*NQO2*	NAD(P)H dehydrogenase, quinone 2	catalyzes the two-electron reduction of quinone substrates and uses dihydronicotinamide riboside as a reducing coenzyme	6.89
*DDIT3*	DNA-damage-inducible transcript 3	member of the CCAAT/enhancer-binding protein (C/EBP) family of transcription factors	4.10
*NQO1*	NAD(P)H dehydrogenase, quinone 1	prevents the one electron reduction of quinones that results in the production of radical species	3.81
*CASP7*	caspase 7, apoptosis-related cysteine peptidase	plays a central role in the execution-phase of cell apoptosis	3.33
*SLC9A1*	solute carrier family 9, subfamily A (NHE1)	plasma membrane transporter	3.00
*DDIT4*	DNA-damage-inducible transcript 4	linked to mTOR signaling, hypoxia response	2.76
*JUN*	jun proto-oncogene	putative transforming gene of avian sarcoma virus 17; protein is highly similar to the viral protein, interacts directly with specific target DNA sequences to regulate gene expression	2.75

**Table 4:** The list of 10 most upregulated genes involved in apoptosis in bortezomib-treated SH-SY5Y cells compared to controls. Gene function according to NCBI Gene database.

Gene Symbol	Gene Name	Gene Function	Fold Change
*HSPA1B*	heat shock 70kDa protein 1B	stabilizes existing proteins against aggregation and mediates the folding of newly translated proteins in the cytosol and in organelles	21.95
*HSPA1A*	heat shock 70kDa protein 1A	similar to *HSPA1B*	20.31
*CLU*	clusterin	involved in several basic biological events such as cell death, tumor progression, and neurodegenerative disorders	17.86
*HMOX1*	heme oxygenase (decycling) 1	cleaves heme to form biliverdin	12.45
*GCLM*	glutamate-cysteine ligase, modifier subunit	first rate limiting enzyme of glutathione synthesis	11.52
*TNFRSF12A*	tumor necrosis factor receptor superfamily, member 12A	cytokine-cytokine receptor interaction	11.18
*SQSTM1*	sequestosome 1	binds ubiquitin and regulates activation of NF-kB signaling pathway	7.12
*GADD45A*	growth arrest and DNA-damage-inducible, alpha	mediates activation of the p38/JNK pathway via MTK1/MEKK4 kinase	6.89
*NQO2*	NAD(P)H dehydrogenase, quinone 2	catalyzes the two-electron reduction of quinone substrates and uses dihydronicotinamide riboside as a reducing coenzyme	6.86
*ATF3*	activating transcription factor 3	member of the mammalian activation transcription factor/cAMP responsive element-binding (CREB) protein family of transcription factors	6.37

**Table 5:** The list of most downregulated genes involved in anti-apoptotic processes in bortezomib-treated SH-SY5Y cells compared to controls. Gene function according to NCBI Gene database.

Gene Symbol	Gene Name	Gene Function	Fold Change
*TFAP2B*	transcription factor AP-2 beta (activating enhancer binding protein 2 beta)	stimulates cell proliferation and suppress terminal differentiation of specific cell types	−13.63
*PDE3A*	phosphodiesterase 3A, cGMP-inhibited	plays critical roles in many cellular processes by regulating the amplitude and duration of intracellular cyclic nucleotide signals	−8.46
*TXNIP*	thioredoxin interacting protein	major regulator of cellular redox signaling which protects cells from oxidative stress	−7.42
*PEG10*	paternally expressed 10	plays a role in cell proliferation, differentiation, and apoptosis	−4.45
*MAP3K1*	mitogen-activated protein kinase kinase kinase 1, E3 ubiquitin protein ligase	a serine/threonine kinase and is part of some signal transduction cascades, including the ERK and JNK kinase pathways as well as the NF-kB pathway	−4.17
*MYC*	v-myc avian myelocytomatosis viral oncogene homolog	encodes a nuclear phosphoprotein that plays a role in cell cycle progression, apoptosis, and cellular transformation	−3.72
*HMGB1*	high mobility group box 1	regulates transcription, and is involved in organization of DNA	−3.33

**Table 6:** The list of most dysregulated miRNAs with their targets, which are involved in neurogenesis, in bortezomib-treated SH-SY5Y cells compared to controls. Presented miRNAs downregulate expression of their target genes, hence the direction of target genes regulation is marked as “↓” and the fold change of target gene is always negative, indicating decreased expression.

miRNA	Fold Change miRNA	Direction of Target Genes Regulation	Target Genes	Fold Change Target Gene mRNA
hsa-miR-1303	7.53	↓	*DCX* (doublecortin)	−2.60
hsa-miR-222-3p	6.33	↓	*CDK6* (cyclin-dependent kinase 6)	−6.75
hsa-miR-192-5p	4.68	↓	*ALCAM* (activated leukocyte cell adhesion molecule)	−4.63
hsa-miR-21-3p	4.13	↓	*NCAM2* (neural cell adhesion molecule 2)	−5.59
hsa-miR-6824-5p	4.12	↓	*SOX4* (sex determining region Y-box 4)	−4.09
hsa-miR-1281	4.00	↓	*DPYSL5* (dihydropyrimidinase-like 5)	−2.27
			*HMGB1* (high mobility group box 1)	−3.33
hsa-miR-335-5p	3.71	↓	*SOX4*	−4.09
hsa-miR-1468-3p	2.86	↓	*SOX4*	−4.09
			*NFIB* (nuclear factor I/B)	−4.30
hsa-miR-34a-5p	2.80	↓	*CDK6*	−6.75
			*MYC* (v-myc avian myelocytomatosis viral oncogene homolog)	−3.72
hsa-miR-8084	2.78	↓	*HMGB1*	−3.33
hsa-miR-2278	2.73	↓	*PPAP2B* (phosphatidic acid phosphatase type 2B)	−2.51
hsa-miR-574-5p	2.56	↓	*DPYSL5*	−2.27
			*CDON* (cell adhesion associated)	−2.52
			*SIGMAR1* (sigma non-opioid intracellular receptor 1)	−2.58
			*SSH2* (slingshot protein phosphatase 2)	−2.39
hsa-miR-6856-5p	2.55	↓	*SV2C* (synaptic vesicle glycoprotein 2C)	−3.14
hsa-miR-3128	2.54	↓	*DPYSL5*	−2.27
hsa-miR-212-3p	2.26	↓	*PTCH1* (patched 1)	−2.35
hsa-miR-6780a-5p	2.21	↓	*DPYSL5*	−2.27
			*NANOS1* (nanos homolog 1)	−2.68
hsa-miR-551b-5p	2.18	↓	*CDK6*	−6.75
hsa-miR-6836-5p	2.17	↓	*CDK6*	−6.75
			*TFAP2B* (transcription factor AP-2 beta (activating enhancer binding protein 2 beta))	−13.63
hsa-miR-4668-5p	2.16	↓	*MYC*	−3.72
			*LYN* (LYN proto-oncogene, Src family tyrosine kinase)	−2.14
hsa-miR-4306	2.11	↓	*PPAP2B*	−2.51
hsa-miR-3192-5p	2.09	↓	*CHRNB2* (cholinergic receptor, nicotinic, beta 2 (neuronal))	−2.03
hsa-miR-572	2.04	↓	*ATM* (ATM serine/threonine kinase)	−2.25

**Table 7:** The list of most dysregulated miRNAs with their pro-apoptotic targets in bortezomib-treated SH-SY5Y cells compared to controls. Presented miRNAs downregulate expression of their target genes, hence the direction of target genes regulation is marked as “↓”. Since all miRNAs have decreased expression (negative fold change), the downregulation of their targets is not prominent and therefore they present with positive fold change, which indicates increased expression of pro-apoptotic genes.

miRNA	Fold Change	Direction of Target Gene Regulation	Target Genes	Fold Change Target Gene mRNA
hsa-miR-330-3p	−3.70	↓	*MDM2* (MDM2 proto-oncogene, E3 ubiquitin protein ligase)	2.43
hsa-miR-6808-5p	−2.81	↓	*ERN1* (endoplasmic reticulum to nucleus signaling 1)	2.22
hsa-miR-124-3p	−2.77	↓	*SNAI2* (snail family zinc finger 2)	5.01
			*EPHA2* (EPH receptor A2)	4.21
			*IL6R* (interleukin 6 receptor)	3.85
hsa-miR-6880-5p	−2.46	↓	*C12orf5* (chromosome 12 open reading frame 5)	2.07
hsa-miR-503-5p	−2.46	↓	*DYNLL2* (dynein, light chain, LC8-type 2)	3.81
			*CDKN1A* (cyclin-dependent kinase inhibitor 1A (p21, Cip1))	2.85
hsa-miR-1226-5p	−2.45	↓	*ARHGEF2* (Rho/Rac guanine nucleotide exchange factor (GEF) 2)	2.04
hsa-miR-500b-3p	−2.37	↓	*DYNLL2*	3.81
hsa-miR-26b-5p	−2.22		*ADAM17* (ADAM metallopeptidase domain 17)	2.49
			*DDIT4* (DNA-damage-inducible transcript 4)	2.76
			*HSPD1* (heat shock 60kDa protein 1 (chaperonin))	2.09
			*EPHA2*	4.21
			*RNF216* (ring finger protein 216)	2.04
hsa-miR-218-5p	−2.04	↓	*RHOB* (ras homolog family member B)	3.01
			*MDM2*	2.43
			*SQSTM1* (sequestosome 1)	7.12
			*FLNA* (filamin A, alpha)	2.24
			*SNAI2*	5.01
			*ADAM17*	2.49

**Table 8:** The list of most dysregulated miRNAs with their targets involved in neuron death, in bortezomib-treated SH-SY5Y cells compared to controls. Presented miRNAs downregulate expression of their target genes, hence the direction of target genes regulation is marked as “↓”. Since all miRNAs have decreased expression (negative fold change), the downregulation of their targets is not prominent and therefore they present with positive fold change, which indicates increased expression of genes related to neuron death.

miRNA	Fold Change miRNA	Direction of Target Gene Regulation	Target Genes	Fold Change Target Gene mRNA
hsa-miR-6880-5p	−2.46	↓	*C12orf5* (chromosome 12 open reading frame 5)	2.07
hsa-miR-26b-5p	−2.22	↓	*DDIT4* (DNA-damage-inducible transcript 4)	2.76
			*HSPD1* (heat shock 60kDa protein 1 (chaperonin))	2.09

**Table 9:** The list of most dysregulated anti-apoptotic miRNAs with their targets, in bortezomib-treated SH-SY5Y cells compared to controls. Presented miRNAs downregulate expression of their target genes, hence the direction of target genes regulation is marked as “↓”. Since all miRNAs have increased expression (positive fold change), the downregulation of their targets is prominent and therefore they present with negative fold change, which indicates decreased expression of anti-apoptotic genes.

miRNA	Fold Change miRNA	Direction of Target Gene Regulation	Target Genes	Fold Change Target Gene mRNA
hsa-miR-22-5p	5.31	↓	*PDE3A* (phosphodiesterase 3A, cGMP-inhibited)	−8.46
hsa-miR-22-3p	4.88	↓	*SERBP1* (SERPINE1 mRNA binding protein 1)	−2.02
hsa-miR-192-5p	4.68	↓	*CADM1* (cell adhesion molecule 1)	−2.52
			*EGR1* (early growth response 1)	−2.89
			*MAP3K1* (mitogen-activated protein kinase kinase kinase 1, E3 ubiquitin protein ligase	−4.17
hsa-miR-21-3p	4.13	↓	*ELMO1* (engulfment and cell motility 1)	−2.30
			*GPAM* (glycerol-3-phosphate acyltransferase, mitochondrial)	−2.36
hsa-miR-6824-5p	4.12	↓	*CHST11* (carbohydrate (chondroitin 4) sulfotransferase 11)	−2.03
			*SOX4* ((sex determining region Y)-box 4)	−4.09
hsa-miR-1281	4.00	↓	*HMGB1* (high mobility group box 1)	−3.33
hsa-miR-335-5p	3.71	↓	*SOX4*	−4.09
hsa-miR-4423-3p	3.28	↓	*IGF1R* (insulin-like growth factor 1 receptor)	−2.28
hsa-miR-4417	3.19	↓	*SATB1* (SATB homeobox 1)	−2.51
hsa-miR-125b-1-3p	3.00	↓	*IGF1R*	−2.28
hsa-miR-1468	2.86	↓	*SOX4*	−4.09
hsa-miR-34a-5p	2.80	↓	*BCL2* (B-cell CLL/lymphoma 2)	−2.77
			*PEG10* (paternally expressed 10)	−4.45
			*MYC* (v-myc avian myelocytomatosis viral oncogene homolog)	−3.72
hsa-miR-8084	2.78	↓	*HMGB1*	−3.33
hsa-miR-3136-5p	2.59	↓	*TXNIP* (thioredoxin interacting protein)	−7.42
hsa-miR-574-5p	2.56	↓	*SATB1* (SATB homeobox 1)	−2.51
			*THRA* (thyroid hormone receptor, alpha)	−2.33
			*SIGMAR1* (sigma non-opioid intracellular receptor 1)	−2.58
hsa-miR-3128	2.54	↓	*PDE3A* (phosphodiesterase 3A, cGMP-inhibited)	−8.46
hsa-miR-3201	2.48	↓	*IVNS1ABP* (influenza virus NS1A binding protein)	−2.14
hsa-miR-551b-5p	2.18	↓	*IGF1R*	−2.28
			*C6orf120* (chromosome 6 open reading frame 120)	−2.56
hsa-miR-6836-5p	2.17	↓	*TFAP2B* (transcription factor AP-2 beta (activating enhancer binding protein 2 beta))	−13.63
hsa-miR-4668-5p	2.16	↓	*MYC*	−3.72
			*SIN3A* (SIN3 transcription regulator family member A)	−2.31
			*LYN* (LYN proto-oncogene, Src family tyrosine kinase)	−2.14
			*GABARAP* (GABA(A) receptor-associated protein)	−2.68
hsa-miR-4306	2.11	↓	*TMEM109* (transmembrane protein 109)	−2.05
			*CADM1*	−2.52
			*CHST11* (carbohydrate (chondroitin 4) sulfotransferase 11)	−2.03
hsa-miR-572	2.04	↓	*ATM* (ATM serine/threonine kinase)	−2.25

## Abbreviations

BSA	bovine serum albumin
DAVID	Database for Annotation, Visualization, and Integrated Discovery
ER	endoplasmatic reticulum
GO	Gene Ontology
miRNAs	MicroRNAs
MM	Multiple myeloma
qRT-PCR	quantitative polymerase chain reaction
RT	room temperature
UPS	ubiquitin/proteasome system
